# Marine Habitat Selection by Marbled Murrelets (*Brachyramphus marmoratus*) during the Breeding Season

**DOI:** 10.1371/journal.pone.0162670

**Published:** 2016-09-28

**Authors:** Teresa J. Lorenz, Martin G. Raphael, Thomas D. Bloxton

**Affiliations:** United States Department of Agriculture, Forest Service, Pacific Northwest Research Station, Olympia, Washington, United States of America; Oregon State University, UNITED STATES

## Abstract

The marbled murrelet (*Brachyramphus marmoratus*) is a declining seabird that is well-known for nesting in coastal old-growth forests in the Pacific Northwest. Most studies of habitat selection have focused on modeling terrestrial nesting habitat even though marine habitat is believed to be a major contributor to population declines in some regions. To address this information gap, we conducted a 5-year study of marine resource selection by murrelets in Washington, which contains a population experiencing the steepest documented declines and where marine habitat is believed to be compromised. Across five years we tracked 157 radio-tagged murrelets during the breeding season (May to August), and used discrete choice models to examine habitat selection. Using an information theoretic approach, our global model had the most support, suggesting that murrelet resource selection at-sea is affected by many factors, both terrestrial and marine. Locations with higher amounts of nesting habitat (*β* = 21.49, *P* < 0.001) that were closer to shore (*β* = -0.0007, P < 0.001) and in cool waters (*β* = -0.2026, *P* < 0.001) with low footprint (*β* = -0.0087, *P* < 0.001) had higher probabilities of use. While past conservation efforts have focused on protecting terrestrial nesting habitat, we echo many past studies calling for future efforts to protect marine habitat for murrelets, as the current emphasis on terrestrial habitat alone may be insufficient for conserving populations. In particular, marine areas in close proximity to old-growth nesting habitat appear important for murrelets during the breeding season and should be priorities for protection.

## Introduction

The marbled murrelet (*Brachyramphus marmoratus*) is a federally threatened seabird that requires two fundamentally different habitats for breeding. Foraging occurs at sea because murrelets are pursuit-diving seabirds whose primary prey are small schooling fish and large zooplankton. However, coastal, old-growth coniferous forests are used for nesting in much of their range. Like many Alcids, marbled murrelets do not build nests and instead rely on naturally deposited materials for a nest platform. Unlike other alcids, however, in the marbled murrelet a single egg is typically laid directly on a large, mossy limb in the forest canopy. In most cases, only large, old trees have limbs of sufficient diameter for these unusual nests.

The first management efforts with marbled murrelets recognized their need for large trees. They also acknowledged that >100 years of logging in coastal forests from British Columbia to California had drastically reduced the amount of murrelet breeding habitat [1]. In the Northwest Forest Plan [2], measures were taken to protect unharvested, late-successional, and old-growth forests on Federal lands in Washington, Oregon, and California. Unfortunately, despite these conservation efforts decades ago, multiple studies have reported continued population declines [3–5]. There has been much discussion in the scientific literature about whether these declines are due to losses in nesting habitat that have continued on private lands and/or changes to marine habitat. Raphael et al. [6] documented an 8–33% decline in potential nesting habitat on private lands from Washington to California which corresponds with murrelet population declines during that same time period [4]. Nest habitat losses were greatest in Washington State, which is where Miller et al. [4] and Falxa and Raphael [5] reported the steepest declines in murrelet numbers. Additionally, marine conditions have also changed in the last century and since the Northwest Forest Plan. Marine factors that have been identified as potential threats to murrelets include overfishing, pollution, unattended fishing gear, human population growth (and associated disturbance), and more recently, climate change [1]. In the southern portion of their range, Peery et al. [7] examined the relative influence of nesting habitat on reproduction in a declining California population and concluded that marine food and nest predation, rather than availability of nesting habitat, were responsible for the low reproductive output of the population. Studies using stable isotopes have indicated that murrelets are foraging on lower trophic levels than historically, and this has contributed to low productivity and population declines [8–10].

Overall, the conservation of marbled murrelets may hinge on protecting not only nesting habitat–the focus of conservation efforts to date–but also on foraging habitat. In an effort to better understand important marine habitats for marbled murrelets, we conducted a study of marine habitat selection by individually-tagged murrelets. Studies of habitat selection by individually tagged animals are important for examining habitat selection for conservation planning [11]. Our objective was to model marine habitat selection by marbled murrelets during the breeding season to better understand the relative influence of marine versus terrestrial habitat features on murrelet space use while at-sea. We conducted this study in northwestern Washington, the location of the steepest murrelet declines documented to date.

## Materials and Methods

### Statement

All handling and tagging of marbled murrelets was in accordance with the U.S. Fish and Wildlife Service Endangered Species 10a1a permit (Permit #TE-070589-2) and in compliance with the Ornithological Council Guidelines for the Use of Wild Birds in Research [12]. Scientific Collection Permits were obtained annually from Washington Department of Fish and Wildlife, and a Federal Bird Banding Permit was obtained from the U.S. Geological Survey, Bird Banding Lab. Field studies involved handling a federally threatened seabird, and consequently all sampling and handling procedures were approved by a U.S. Fish and Wildlife Service Endangered Species 10a1a permit prior to the start of our study, as noted above. Permissions to access field sites were provided by United States (U.S.) Forest Service, U.S. Park Service, Washington Department of Fish and Wildlife, Washington Department of Natural Resources, Washington State Parks, and British Colombia Ministry of Forests, Lands, and Natural Resource Operations.

### Field methods

**Fig 1:**
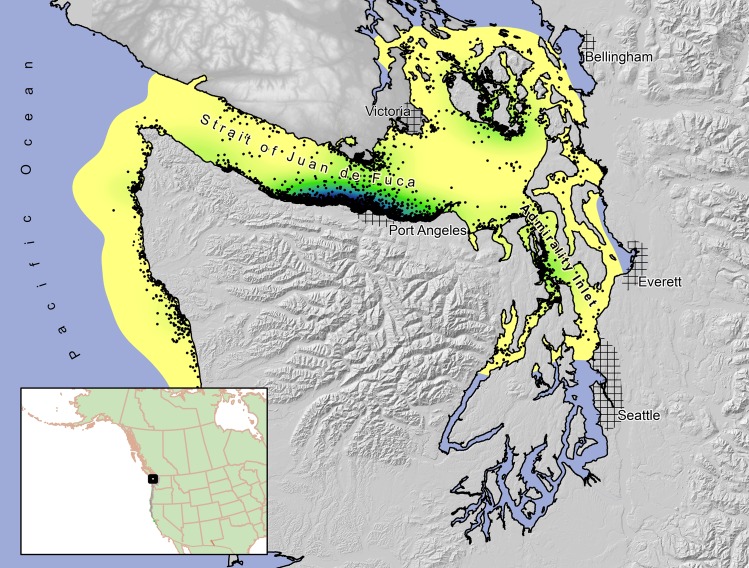
Study area used to examine resource selection by marbled murrelets in northwestern Washington and southwestern British Columbia, 2004–2008. The marine 99% population-level kernel (from all radio-tracked murrelets) is depicted in yellow to green tones, where darker shading indicates areas with a high probability of use and lighter yellow shading areas with a low probability of use by the population of tagged murrelets. Black dots represent 5,388 marine telemetry locations from all murrelets tracked in this study.

We conducted this study in northwestern Washington State (approximately 47° 48′ N, 123° 40′ W) and southwestern British Columbia (approximately 48° 24′ N, 123° 40′ W) (Fig 1). We were logistically constrained to capturing birds in U.S. waters, although we radio-tracked murrelets in both U.S. and Canadian waters. We used standard techniques to capture murrelets at-sea, which involved locating birds at night from small boats using night-lighting and capturing them in long-handled dipnets [13]. We captured and tagged murrelets from April to July, 2004–2008. With the exception of a few individuals that were released without transmitters because of concerns over handling stress, all birds were fit with a VHF transmitter (1.5% of body weight; Advanced Telemetry Systems, Isanti, MN) using a subcutaneous anchor following Newman et al. (1999) [14]. Unlike Newman et al. [13] however, we did not use anesthesia or sutures. Murrelets were released at the site of capture within 1 hour.

We located radio-tagged murrelets using aerial tracking from fixed wing aircraft. We initiated searches within three days after the first murrelet was tagged in each year. We ended searches after the last known nest had fledged or failed and when significant numbers of transmitters were no longer detectable within our study area, indicating post-breeding dispersal or transmitter battery failure. Weather permitting, we conducted tracking flights daily. Tracking flights lasted for up to ~5 hours until either all birds had been located or the aircraft needed refueling. Aerial searches focused on marine areas, but also included terrestrial areas to locate nest sites of breeding murrelets. If an individual murrelet was not located at-sea or on an inland nest for ~2–3 consecutive days, we expanded our search area to find the missing bird and generally focused on areas beyond the location that the missing murrelet was last detected.

When a murrelet’s radio signal was detected from the air, pilots circled over the transmitter and used a GPS unit to mark the location from which they heard the loudest radio signal. Tests with stationary transmitters indicated that location accuracy from aircraft was 385 m on average (SD = 230, range 93–685 m). We omitted all telemetry locations obtained at night (defined by civil twilight on each day) because past research indicates murrelets do not actively feed at night [15] and we were primarily interested in habitat selection by active and foraging murrelets.

### Habitat data

Based on a review of the literature and our own observations, we considered 12 habitat variables influential in marine space use (Table 1). Marbled murrelets forage on a variety of invertebrate and vertebrate prey including sand lance (*Ammodytes hexapterus*), anchovy (*Engraulis mordax*), herring (*Chulpea harengus*), smelt (*Osmeridae*), surfperch (*Cymatogaster aggregate*), juvenile rockfish (*Scorpaenidae*), and krill (*Euphausiids*) [16]. Spatially explicit information on the distribution of these prey were not available for our area, but remotely sensed marine conditions are often associated with the distribution of fish and krill ([16–21]; but see [22–23]). In past studies, fish distributions have been associated with remotely sensed sea surface temperature (SST; cooler temperatures generally favor increased prey; e.g., Emmett et al. [19]) and chlorophyll-*a* concentrations (higher levels generally associated with higher prey; e.g., Peterson et al. [20]). We obtained satellite derived data on SST and chlorophyll-*a* for each month and year of our study from the National Oceanic and Atmospheric Administration [24]. We obtained data on diffuse attenuation as an estimate of water turbidity, which can affect murrelet [25] and fish abundance [27]. These remotely sensed data were obtained from Aqua MODIS (Moderate Resolution Imaging Spectroradiometer) at a resolution of 1 km (SST and chlorophyll-*a*) and 4 km (diffuse attenuation). We considered the effect of both current- and previous-month SST and chlorophyll-*a* on murrelet space use to account for possible time-lags between these factors and responses of the murrelet’s prey.

**Table 1:** Description of parameters considered for examining marine habitat selection by marbled murrelets in northwestern Washington, USA, 2004–2008.

Parameter	Description	Source	Examples of previous research that has suggested or found parameter to be influential^1^
Shoredist	Distance to shore (m)	Washington Department of Natural Resources	Ralph et al. [55], Day et al. [25], Raphael et al. [26], and others
Depth	Water depth (bathymetry; m)	U.S. Geological Survey	Barrett [35], Ralph et al. [55], Day et al. [25], and others
PreviousSST	Previous month sea surface temperature (C)	NOAA 2007	Raphael et al. [26]
CurrentSST	Current month sea surface temperature (C)	NOAA 2007	Becker and Beissinger [41], Day et al. [25], Barrett [35], and others
PreviousChlor	Previous month chlorophyll-a (mg/m^3^)	NOAA 2007	Raphael et al. [26]
CurrentChlor	Current month chlorophyll-a (mg/m^3^)	NOAA 2007	Miller et al. [37], Raphael et al. [26], and others
Turbidity	Diffuse attenuation (0.1 m)	NOAA 2007	Day et al. [25], Renner et al. [21]
Marinefoot	Marine human footprint (scale of 0–100)	Halpern et al. 2015	Speckman et al. [27], Bellefleur et al. [28], Raphael et al. [29]
Terrestialfoot	Terrestrial human footprint (scale of 0–100)	Sanderson et al. 2002	Raphael et al. [26]
Wind	Wind Power Class (categorical variable: low or high)	NREL 2012	Sealy [31]
Shoretype	Shoreline composition (categorical variable: sand/gravel beach or other)	NOAA 2002 and BC Ministry of Forests, Lands and Natural Resource Management	Yen et al. [34], Barrett [35]
Nesthabitat	Proportion of area within 43 km classified as nesting habitat	Raphael et al. 2011, 2014	Miller et al. [37], Becker and Beissinger [41], and others

^1^Literature citations are provided as examples of studies that have found variables influential in marbled murrelet ecology; the list is not meant to be exhaustive or all-inclusive.

We measured the linear distance from each telemetry relocation to the shore. We also measured water depth (bathymetry) at each relocation point because murrelets are thought to forage in shallow water [26]. Human activity can affect marbled murrelet space use at-sea [27–28] and we therefore included indices of marine and terrestrial human footprint for our study area. The marine footprint was obtained from Halpern et al. [29] and the terrestrial footprint from Sanderson et al. [30]. The marine footprint combined 17 factors ranging from fishing activity, pollution, and shipping traffic [29]. The terrestrial footprint considered three main factors: human population density, light pollution, and transportation infrastructure (including roads, railways, coastlines, and rivers) [30]. Both datasets were calculated at ~1 km resolution and classified the marine or terrestrial landscape on a scale of 0–100 in the relative influence of human activity.

We included a categorical variable for the average relative wind speed (or “windiness”) because murrelets may preferentially foraged in areas of calm water [31]. We obtained spatial data on estimated wind energy potential (i.e., Wind Power Class) at 500 m resolution from the National Renewable Energy Laboratory [32] and used this as a proxy for the relative wind speed on waters within our study area. Based on this dataset [32] we classified windiness within our study area as a dichotomous variable (high or low) where high winds were associated with areas with a Wind Power Class ≥3 (areas suitable for wind energy development, with annual average wind speed at heights of 50 m > 6.4–7.0 m/s) and low winds in areas with a Wind Power Class <3 (areas unsuitable for wind energy development, with annual average wind speed at heights of 50 m < 6.4–7.0 m/s) [32].

Sand lance (*Ammodytes hexapterus*) are considered one important prey of breeding marbled murrelets ([16], and references therein; [33]). They are associated with fine gravel or sandy-bottomed coastal waters and thus marbled murrelets may select sandy bottomed areas over rock or other substrates. We could find no spatial data on bottom types for our study area and so we used the nearest shoreline type as a proxy [34–35]. We obtained spatial data on shoreline composition from the National Oceanic and Atmospheric Administration for U.S. shorelines [36] and British Columbia Ministry of Forests, Lands and Natural Resource Management for Canadian shorelines. We then classified the entire shoreline of our study area into two classes, “sand/gravel beach” and “other” shoreline. Our class for “sand/gravel beach” included sand and mixed- gravel beaches. Our class for “other” included shores classified as rock shoreline (cliff to level, rocky shores), human structures, and vegetated, tidal wetlands.

The availability and proximity of potential nesting habitat has been implicated as an important factor affecting the marine density of murrelets in multiple studies [26, 37]. We estimated the proportion of nesting habitat within a 5806-km^2^ circular area centered on each telemetry location, equal in radius to the mean distance traveled to sea for breeding murrelets in our study. We obtained spatial data on suitable nesting habitat for our study area from Raphael et al. [6, 26]. For the U.S. portion of our study area, Raphael et al. [6] defined nesting habitat primarily using LandTrendr data (Landsat-based detection of Trends in Disturbance and Recovery methods) [38] and Gradient Nearest Neighbor (GNN) models [39]. For the Canadian portion of our study, nesting habitat was defined based on areas classified as Old Growth Management areas by the Ministry of Forests, Lands, and Natural Resources [40]. While this dataset does not explicitly model nest habitat for murrelets, it was the most recent and comprehensive layer for potential nesting habitat that we were able to obtain, and has been used in other publications modeling murrelet nesting habitat availability [26].

We did not consider distance to nest site as a factor [41–42] because only a few radio-tagged murrelets in our study were confirmed breeders with known nest sites. However, by including the proportion of nesting habitat as a factor, we assumed that the effect of nest site proximity and availability was adequately accounted for in our analysis. Also, we did not differentiate between breeders and nonbreeders in our analysis because few murrelets bred (13%). Because murrelets commonly visit nesting habitat even when not actively breeding [43–44] we did not exclude proportion of nesting habitat from our analysis for non-breeders. We expected that availability of nesting habitat had the potential to influence murrelet marine space use regardless of breeding status. We did not differentiate between males and females in our analysis because past studies have found that marine movements and space use do not vary by sex [42, 45], which is supported by observations of murrelets associating as pairs while at-sea during the breeding season [46]. We did not consider some factors that had poor support in past studies and which exploratory analyses indicated were not influential in our study, including distance to nearest river [26, 35], distance to kelp beds [47–48], and underwater slope [35].

### Defining availability

Ocean conditions were in a continual state of flux during our study and therefore we used discrete choice models to examine resource selection by marbled murrelets. Discrete choice models are appropriate for dynamic systems in which availability changes because used resources are compared only to available resources within a “choice set” that is unique to each used location. In other words, availability is defined separately for each used resource unit and therefore more accurately reflects spatial and temporal variability in resources. Thus, we were able to examine selection for marine resources even as ocean conditions changed over the months and years of our study.

We defined availability using a circular buffer centered on each used point equal to 46-km radius, which represented the 95^th^ percentile of daily step lengths by all murrelets in our study. Within these circular buffers, we further restricted availability to marine areas (we excluded land and freshwater lakes) that occurred within the 99% population fixed kernel utilization distribution estimated from Geospatial Modeling Environment software [49]. Most of the areas excluded by using this restricted definition of availability were far-offshore (e.g., >20 km). All research to date indicates that such pelagic environments are rarely or never used by marbled murrelets [50–51] (and murrelets were not detected >10.9 km offshore in the course of our study) and we assumed that including them as available habitat would bias our estimates of resource selection.

To represent availability, we generated 5 random points within each circular buffer following recommendations by Baasch et al. [52]. We then used ArcGIS 10 (Environmental Systems Research Institute, Inc., Redlands, CA) to extract remotely sensed habitat data for each used and available point within a choice set. Because of the relatively low spatial resolution of some raster data layers and error associated with telemetry locations obtained from the air, we also considered using error polygons around telemetry locations to characterize habitats. For error polygons, we averaged habitat characteristics within different sized error polygons centered on each used location. We compared habitat composition between telemetry relocation point data (e.g., 0-m radius buffer) and then 500-m radius buffers, 1000-m radius buffers, 1500-m radius buffers centered around telemetry relocation points. When determining habitat attributes within buffers of 500- to 1500-m radius, we averaged habitat attributes for all grid cells contained within the buffer area. We observed high correlations among each of those four datasets for all habitat factors that we compared (r > 0.95). For simplicity, we therefore used habitats characteristics estimated from point data in our analysis.

### Analysis

We used an information-theoretic approach [53] to develop candidate models explaining murrelet marine habitat selection. We built a set of a priori models based on published literature and our own observations of murrelet marine space use. Prior to building models, we first considered whether some variables were better modeled by quadratic rather than linear effects [54]. Based on the ecology of the marbled murrelet, factors that we thought may be better modeled using a quadratic term were sea surface temperature, chlorophyll-*a*, and turbidity [25, 35]. To determine whether these factors should be included as quadratics, we compared AIC_c_ values for univariate models, first considering a model with only the linear term and then only the quadratic term for each of those three factors. We selected the associated form of the covariate from the model with the lowest AIC_c_ (either linear or quadratic) and included it in further analysis. For all other factors, we did not consider a quadratic relationship; prior research indicated that murrelets should select areas with low human footprint and high amounts of nesting habitat that were close to shore and in shallow waters [26, 27, 55]. We also considered a random effect for each individual bird to account for repeated observations and varying sample sizes per bird.

Prior to building our models we assessed possible correlations between pairwise combinations of covariates with the intention of omitting covariates if their coefficient >0.7, [56]. However, no pairwise comparisons had a correlation coefficient >0.7 and therefore none were omitted. We then built candidate models that considered the potential effects of habitat covariates on marine space use (Table 1) based on published literature and our observations of murrelet space use, and we limited our candidate set to fewer than 20 models [57]. Because the selection of models requires some subjectivity, we used also used a variable ranking approach to ascertain the most influential variables from within our top model (see below).

We used Akaike’s Information Criterion corrected for small sample sizes (AIC_c_) to assess the amount of support for different models and considered the model with the lowest AIC_c_ as the best supported given our data. We evaluated whether including a random effect for each individual bird improved model fit by comparing AIC_c_ values for all models with and without a random effect. We also considered whether an interaction term for distance-to-shore and terrestrial human footprint should be included in our top model, because we thought that murrelet use of the shoreline may hinge upon the amount of human disturbance in the area; i.e., murrelets may prefer areas close to shore only where human disturbance is low. Therefore, we compared AIC_c_ values for our top model with an additive and then multiplicative relationship between these two variables [58], and considered the model with the lower AIC_c_ value as having more support.

To assess goodness-of-fit for our top model, we used a modified *k*-fold approach for discrete choice models [59–60]. We partitioned our data into 10 sets of test data that each contained 10% of all choice sets. We then fit a model using the remaining training data associated with each test set (i.e., the entire dataset minus test data associated with each training dataset). We then tested the ability of each training model to correctly predict used locations within choice sets in the test data. Sites predicted as “used” were those with the highest predicted probability of use in each choice set. We calculated the percentage of choice sets in which selection was correctly assigned and averaged the percentage across all 10 test/training sets. Based on random chance alone we expected 17% (1 in 6) of used sites to be correctly identified, and values >17% suggested that our model predicted use better than random.

We assessed the relative importance of our habitat variables in the best-supported model using two methods. First, we computed parameter estimates using standardized variables and then ranked variables based on the absolute value of the coefficients. This method compares the effect of a one standard deviation change in each variable on the dependent variable (relative probability of selection). Second, to evaluate the numerical influence of each explanatory variable on selection, we set all explanatory variables except one to their mean values. We then noted the change in the probability of selection for values of that one variable set to its maximum and minimum value. We ranked variables based on the numerical change they caused to the dependent variable.

We used R version 3.1.1 [61] for all statistical analyses and fit discrete choice models using the coxme and mclogit packages. We report means with their standard deviation unless otherwise noted.

## Results

### Telemetry tracking

We captured, tagged, and radio-tracked 157 murrelets from 2004 to 2008. Twenty (13%) radio-tagged murrelets attempted to breed and we estimated that 4 successfully fledged young. Breeding murrelets traveled on average 43.3 km from nest sites to used marine locations. For our analysis of habitat selection, we therefore used a buffer distance of 43 km for estimating the proportion of nesting habitat near marine locations.

Across all tagged murrelets, we obtained 5,388 diurnal marine relocations during telemetry tracking flights. We considered consecutive telemetry relocations to be independent because they were obtained on different days (i.e., one point per day). We omitted 33 individuals from our analysis that had <20 marine telemetry relocations and retained 124 birds to examine habitat selection. On average, we obtained 39 diurnal marine relocations (SD = 11 relocations; range 20–69) on these 124 individuals for a total of 5,185 used locations for discrete choice modeling.

**Fig 2:**
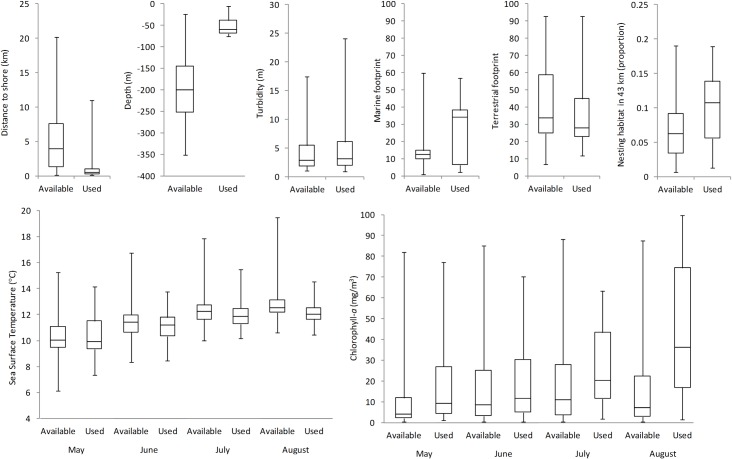
Comparison of habitat features at locations available versus used by marbled murrelets in northwestern Washington, U.S.A., and southwestern British Columbia, Canada, 2004–2008. Box plots show maximum (top whisker), minimum (bottom whisker), first and third quartiles (IQR; top and bottom line of box), and median (center line of box).

On average, murrelets foraged 0.95 km (95% CI = 0.92–0.98 km) from shore in waters 30.3 m deep (SD = 35.9 m). Across our study area, SST ranged between 6.1 and 19.5°C over the course of our study, increasing as summers progressed (Fig 2). Waters used by murrelets were similar in temperature on average ($\bar{x}=10.1$, SE = 0.02°C in May; $\bar{x}=12.2$, SE = 0.01°C in August) to those available within the study area ($\bar{x}=10.3$, SE = 0.8°C in May; $\bar{x}=12.8$, SE = 0.8°C in August). As waters warmed over the summer, used areas showed slightly less variability in temperature compared to available areas (Fig 2). Areas used by murrelets had marginally higher levels of chlorophyll-*a* than available areas (Fig 2), and this effect increased as the season progressed; by August, used areas averaged 44.7 mg/m^3^ (SE = 0.43 mg/m^3^) compared to available areas ($\bar{x}=17.1$, SE = 0.14 mg/m^3^). Nesting habitat at used areas was slightly greater than at available locations (Fig 2). Within 43 km of used telemetry locations, 10.0% (SE = 0.07%) of the landscape was classified as suitable for nesting, compared to available locations, which had 6.8% (SE = 0.02%) of the land area classified as suitable.

### Marine resource selection

AIC_c_ values indicated that murrelet selection of marine areas was better modeled by a linear relationship for SST and turbidity, and by a quadratic relationship for chlorophyll-*a*. When comparing an additive versus multiplicative relationship between shore distance and terrestrial footprint, the multiplicative relationship (interaction) had more support. Including a random effect for individual birds did not substantially improve model fit; the difference in AIC_c_ for eight models, including our top model, with and without a random effect was ≤2 (Table 2). For six models, the addition of a random effect cause the model to fail to converge and for parsimony we therefore did not include a random effect for individual birds in our final model.

**Table 2:** Support for models explaining marine resource selection by marbled murrelets in northwestern Washington, U.S.A., and southwestern British Columbia, Canada, 2004–2008.

Model	*k*	AIC_c_ without random effect	Δ_*i*_	*w*_*i*_	AIC_c_ with random effect
Global with interaction	15	8456	0	0.99	8458
Global with no interactions	14	8476	20.41	<0.01	did not converge
Shoredist, terrestrialfoot, depth, nesthabitat	4	8823	367.2	<0.01	8825
Shoredist, terrestrialfoot, nesthabitat	3	8894	438.4	<0.01	did not converge
Shoredist, terrestrialfoot, depth	3	9874	1418	<0.01	did not converge
Shoredist, terrestrialfoot, shoretype	3	10084	1628	<0.01	did not converge
Wind, depth	2	11375	2920	<0.01	11378
CurrentChlor, shoretype, depth	4	12084	3628	<0.01	12086
Depth	1	12169	3713	<0.01	12171
PreviousChlor, previousSST, currentChlor, currentSST, wind, shoretype, turbidity	9	13725	5269	<0.01	13727
CurrentSST, nesthabitat	2	13729	5273	<0.01	13731
Nesthabitat	1	14057	5601	<0.01	14059
Terrestrialfoot, marinefoot	2	14847	6391	<0.01	did not converge
PreviousChlor, previousSST	3	16105	7649	<0.01	did not converge
CurrentChlor, currentSST	3	16176	7721	<0.01	16178
CurrentSST	1	16938	8482	<0.01	10086

**Fig 3:**
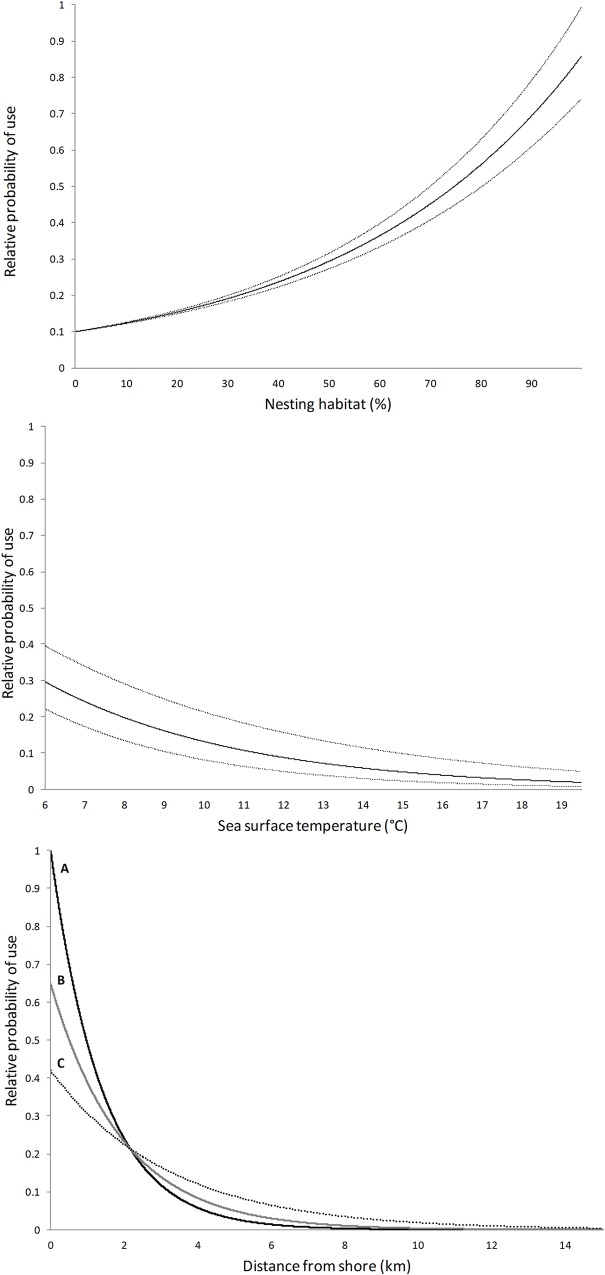
Predicted relative probability, with 95% confidence intervals, of a marine area being used by marbled murrelets in Washington, U.S.A., and southwestern British Columbia, Canada, 2004–2008. Plots show relative probability of use for percent nesting habitat within 43 km (top), and SST (middle), and relative probability as a function of distance to shore (bottom) for areas with low (A; 0), medium (B; 50), and high (C; 100) terrestrial human footprint.

**Fig 4:**
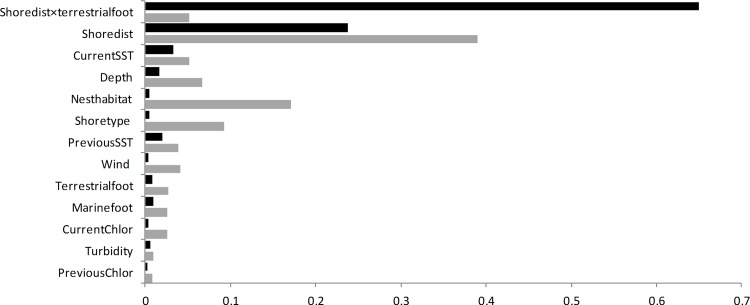
Relative influence of variables explaining marine resource selection marbled murrelets in northwestern Washington, U.S.A., and southwestern British Columbia, Canada, 2004–2008. Black bars indicate relative influence based on effect of each variable on the numerical change in the response variable (holding other variables constant) whereas gray bars indicate relative influence based on ranking of standardized regression coefficients.

The model with the strongest support was the global model with an interaction term for terrestrial footprint and distance-to-shore (Table 2). Ten-fold cross-validation showed that on average, our model correctly predicted use in 59% of cases. Parameter estimates indicated that while murrelets in general prefer areas close to shore, they selected marine locations that were further from shore where the terrestrial footprint was high (Table 3). Nearshore areas with a low terrestrial footprint (e.g., terrestrial footprint ≈ 0) had more the double the predicted probability of use compared to areas with high terrestrial footprint (e.g., terrestrial footprint ≈ 100; Fig 3). Within this near-shore environment, murrelets also selected locations with higher proportional area of nesting habitat and in cooler, calmer, and shallower waters than random sites (Table 3). While chlorophyll-*a*, turbidity, and marine footprint were in our top model, variable importance rankings indicated that they consistently had a relatively small effect on murrelet use of marine environments (Fig 4).

**Table 3:** Parameter estimates, unconditional standard errors, p-values, and 95% confidence intervals for the best-supported model explaining marine resource selection by marbled murrelets in northwestern Washington, U.S.A., and southwestern British Columbia, Canada, 2004–2008.

Parameter	Estimate	Upper CI	Lower CI
CurrentChlor	-0.0081	-0.0020	-0.0142
CurrentChlor^2^	0.0001	0.0001	<0.0001
CurrentSST	-0.2026	-0.1544	-0.2507
Depth	0.0057	0.0070	0.0044
Marinefoot	0.0111	0.0141	0.0081
Nesthabitat	21.49	22.95	20.03
PreviousChlor	0.0045	0.0107	-0.0017
PreviousChlor^2^	-0.0001	<0.0001	-0.0001
PreviousSST	-0.1565	-0.1024	-0.2106
Shoretype	-0.5048	-0.4112	-0.5985
Shoredist	-0.0007	-0.0006	-0.0008
Shoredist×terrestrialfoot	0.000004	0.000006	0.000002
Terrestrialfoot	-0.0087	-0.0052	-0.0122
Turbidity	0.0630	0.1160	0.0099
Wind	0.2250	0.3543	0.0957

When ranking variables by standardized coefficients, the two most influential variables were distance to shore and nesting habitat. Our second ranking method (numerical influence of variables on selection) indicated that SST, distance to shore and its interaction with terrestrial footprint were most influential. Considering the effect of these variables collectively, the predicted probability of a marine area being used by a murrelet increased as nesting habitat increased and distance to shore, SST, and terrestrial human footprint decreased (Fig 4). Most near-shore areas showed a high probability of use relative to open water >2 km off-shore. Exceptions include areas with an extremely high terrestrial footprint, such as the coastline on the far eastern portion of Puget Sound, where Everett and Seattle metropolitan areas are located.

## Discussion

The spatial distribution of murrelets at sea was best explained by our global model, suggesting that multiple factors have some effect on marine space use. All analyses indicated that both marine and terrestrial factors pay significant roles in determining the use of marine habitats. Distance-to-shore, a terrestrial footprint/distance-to-shore interaction, and nesting habitat availability were the three most influential factors. In past studies, distance-to-shore and nesting habitat availability have been shown important in driving murrelet space use or densities. Murrelets are associated with marine areas close to shore and near larger and more cohesive tracts of potential nesting habitat [48, 50–51, 62–64]. The impact of terrestrial human footprint on murrelets has been less studied. The only other study we could find that considered the impact of terrestrial footprint on murrelet occurrence at-sea was Raphael et al. [26]. For areas that are sparsely populated, like coastal Alaska, terrestrial human activities are unlikely to have an impact because human population density is low. For more densely populated areas, however, human activities on land may impact murrelets, and terrestrial footprint should be examined more thoroughly in future studies. Possible direct mechanisms by which terrestrial footprint may impact murrelets is via increased boat traffic in nearby waters (e.g., [27, 28]) or noise disturbance (e.g., [65]). Terrestrial footprint may indirectly impact murrelets if areas with higher footprint are associated with higher amounts of pollution or runoff that reduce prey abundance, or by reductions in nesting habitat.

Influential variables that ranked slightly lower in our analysis included shore type and sea surface temperature (SST). Both these factors serve as proxies for potential murrelet prey and may indicate that murrelets were selecting habits with higher abundance of food [34–35, 41, 48]. Sand and gravel beaches are used as a proxy to indicate the presence of sand lance habitat [66–69], which are prey for murrelets [16, 33]. Cooler sea surface temperatures may point to potentially productive marine conditions that attract seabird prey in general, and may lead to selection by seabirds, increased abundance of seabirds and/or improve seabird reproductive success [23, 70–72].

To verify these suppositions, however, information is needed on the distribution and abundance of murrelet prey in this region. Responses of prey to marine habitats or conditions can be complex. For example, within our study area, the few data available on sand lance abundance suggest that sand lance occur in most (78%) shallow or nearshore waters in the Salish Sea [73]. Thus, our assumption that that sand lance were limited to sand and gravel beaches (and murrelets in our study therefore selected waters near beaches) may have been an oversimplification [69]. Additional data of higher spatial and temporal resolution are needed on sand lance distributions with this region.

The impact of SST on prey is also complex. Several trophic levels can occur between phytoplankton–the trophic level that responds directly to factors like SST–and the prey used for forage by top marine predators like the marbled murrelet. Relationships between murrelets and SST have been variable and scale dependent in some past studies (e.g., [35, 41]). Additionally, there have been situations in past studies where proxies of prey were not associated with actual prey. For example, Gremillet et al. [22] observed that gannets (*Morus capensis*) foraged in areas with predicted high primary productivity (e.g., low SST and high chlorophyll-*a*), but these areas lacked fish. Thus, there was a mismatch between ocean indicators of primary productivity and the prey required for the seabirds. This led to low seabird reproductive output. The seabirds were limited by the availability of nesting habitat (islands) that forced them to nest near areas with poor food resources. With these considerations in mind, we encourage future studies that determine the actual distributions of murrelet prey in our study area. We also encourage studies that map the proximity of productive marine areas relative to suitable nesting habitat. If the proximity of nesting habitat to regions with high food production is important for the marbled murrelet, than measures to protect and enhance nesting habitat near productive marine areas should be prioritized [74]. The conservation of most nesting habitat so far has been based on landownership. In the Northwest Forest Plan, perhaps the most comprehensive habitat protection program to date, only nesting habitat in Federal National Parks and Forests was protected. Potential nesting habitat in adjacent state and private forests, which often occur closer to marine areas, was not protected. Therefore, protection of some nesting habitat in the U.S.A. has been done without full consideration of the habitat needs of nesting murrelets.

### Study limitations

It is important to consider that telemetry location errors in our study had the potential to cause misclassification of some used habitats [75]. We determined that error polygons (from telemetry locations) averaged 385 m, but ranged up to 685 m, which is greater than the 500-m resolution of some of our habitat layers. Other errors may have occurred for used points that occurred near the boundaries of different habitats [74–76]. Thus, some used points may have been placed within habitats that were not actually used. While we found little difference in habitat use when comparing habitats averaged within error polygons to those from point data (correlations >0.95), we nevertheless encourage future studies that minimize location errors to the extent possible. Another equally important consideration is that habitats selected at finer scales, like those considered in our study, depended on habitat selection by murrelets at broader scales [41, 77–78]. Therefore, the results of our study should be applied only within the context of studies that have occurred at broader spatial scales in this region. Studies at larger spatial scales have shown the importance of inland nesting habitat on murrelet marine abundance [26, 77]. Our analysis was restricted to relatively fine spatial scales, and this may explain why nesting habitat availability was not more strongly selected in our study (ranking 2^nd^ and 9^th^ in our two variable rankings) compared to other studies.

Lastly, we were unable to determine the behavior of murrelets that were being tracked, which is important for determining whether used habitats are beneficial to animals under study [79–80]. Murrelets may not have been actively or successfully feeding while using the habitats that we recorded. Identifying murrelet behavior while at-sea may be logistically difficult in some cases without considerable advances in technology, although past studies have used murrelet diving behavior (measured from land using radio telemetry) to infer foraging effort [6, 81]. We did not include information on diving behavior in our analysis, however, and interpretations about the importance of marine habitats for murrelets should be done with our study’s limitations in mind.

### Management implications

Many past studies have called for the protection of marine areas for marbled murrelets, in addition to the measures currently in place to protect terrestrial nesting habitat [9, 74, 82]. Given the marine habitat selection we observed in this study, we suggest that marine areas that should be prioritized for protection are those in closest proximity to large tracts of nesting habitat, with low human footprint, and near sand or gravel beaches. We also suggest that future efforts consider protecting nesting habitat on non-federal lands, as private and state-owned lands often occur in closer proximity to marine areas, at least in northwestern Washington. If murrelets prefer marine areas near nesting habitat, such protections may improve the suitability of some nearshore marine areas for murrelets.

To further guide management efforts, we also suggest that an important next step is to obtain spatially and temporally explicit information on the distribution of forage fish and other murrelet prey in our study area. Such research should also examine the distributions of forage fish and marine invertebrates relative to remotely sensed SST and chlorophyll-*a* in Washington. Information on the distribution of forage fish would aid in assessing the extent to which remotely sensed variables can be used to predict murrelet space use or abundance in our study area. Lacking such information, our results suggest murrelet use of some near-shore areas may be limited by nesting habitat availability, shoreline type, and terrestrial footprint, particularly around large metropolitan areas. Future efforts should explore this further, with the possibility of protecting shoreline near the remaining tracts of nesting habitat from a high degree of development. While we recognize the challenges posed by such an approach, we contend that successful conservation of the marbled murrelet is likely to involve challenges irrespective of the approach taken.

## Supporting Information

S1 Table Data on marbled murrelet use of marine habitats in northwestern Washington, U.S.A., and southwestern British Columbia, Canada, 2004–2008, compared to available habitats. (XLSX)

## References

[1] USFWS (U.S. Fish and Wildlife Service). Recovery plan for the threatened Marbled Murrelet (Brachyramphus marmoratus) in Washington, Oregon, and California. USFWS, Portland, Oregon. 1997.

[2] U.S. Department of Agriculture, Forest Service, U.S. Department of the Interior, Bureau of Land Management. 1994. Record of decision for amendments to the Forest Service and Bureau of Land Management planning documents within the range of the northern spotted owl. 74 p.

[3] Strong CS. Decline of the marbled murrelet population on the central Oregon coast during the 1990s. Northwestern Naturalist. 2003; 84: 31–37.

[4] Miller SL, Raphael MG, Falxa GA, Strong C, Baldwin J, Bloxton T, et al Recent population decline of the marbled murrelet in the Pacific Northwest. Condor. 2012; 114: 771–781.

[5] Falxa GA, Raphael MG. Northwest Forest Plan—The first 20 years (1994–2013): status and trend of marbled murrelet populations and nesting habitat. Gen. Tech. Rep. PNW-GTR-933. Portland, OR: U.S. Department of Agriculture, Forest Service, Pacific Northwest Research Station. 2016.

[6] Raphael MG, Falxa GA, Dugger KM, Galleher BM, Lynch D, Miller SL, et al. Status and trend of nesting habitat for the marbled murrelet under the Northwest Forest Plan. General Technical Report. PNW-GTR-848. U.S. Department of Agriculture, Forest Service, Portland, OR. 2011.

[7] Peery MZ, Beissinger SR, Newman SH, Burkett EB, Williams TD. Applying the declining population paradigm: diagnosing causes of poor reproduction in the marbled murrelet. Conservation Biology. 2004; 18: 1088–1098.

[8] Becker BH, Beissinger SR. Centennial decline in the trophic level of an endangered seabird after fisheries decline. Conservation Biology. 2006; 20: 470–479. 16903108 10.1111/j.1523-1739.2006.00379.x

[9] Norris DR, Arcese P, Preikshot D, Bertram DF, Kyser TK. Diet reconstruction and historic population dynamics in a threatened seabird. Journal of Applied Ecology. 2007; 44: 875–884.

[10] Gutowsky S, Janssen MH, Arcese P, Kyser TK, Ethier D, Wunder MB, et al Concurrent declines in nestling diet quality and reproductive success of a threatened seabird over 150 years. Endangered Species Research. 2009; 9: 247–254.

[11] Millspaugh JJ, Marzluff JM. Radio tracking and animal populations Academic Press, San Diego, CA 2001.

[12] Fair J, Paul E, Jones J. Guidelines to the Use of Wild Birds in Research Ornithological Council, Washington, DC 2010.

[13] Whitworth DL, Takekawa JY, Carter HR, McIver WR. Night-lighting as an at sea capture technique for Xantus’ murrelets in the southern California Bight. Colonial Waterbirds. 1997; 20: 525–531.

[14] Newman SH, Takekawa JY, Whitworth DL, Burkett EE. Subcutaneous anchor attachment increases retention of radio transmitters on Xantus’ and marbled murrelets. Journal of Field Ornithology. 1999; 70: 520–534.

[15] Haynes TB, Nelson SK, Newman SH. Diel shifts in the marine distribution on marbled murrelets near Port Snettisham, Southeast Alaska. Waterbirds. 2010; 33: 471–478.

[16] Burkett EE. Marbled murrelet food habits and prey ecology. In: Ralph CJ, Hunt GL, Raphael MG, Piatt JF, editors. Ecology and conservation of the marbled murrelet. General Technical Report. PSW-GTR-152. U.S. Department of Agriculture, Forest Service. Pp. 223–246. 1995.

[17] Brodeur RD, Fisher JP, Emmett RL, Morgan CA, Casillas E. Species composition and community structure of pelagic nekton off Oregon and Washington under variable oceanographic conditions. Marine Ecology Progress Series. 2005; 298: 41–57.

[18] Emmitt RL, Brodeur RD, Miller TW, Pool SS, Krutzikowsky GK, Bentley PJ, McCrae J. Pacific sardine (*Sardinops sagax*) abundance, distribution, and ecological relationships in the Pacific Northwest. California Cooperative Oceanic Fisheries Investigations Report. 2005; 46: 122–143.

[19] Emmett RL, Krutzikowsky GK, Bentley P. Abundance and distribution of pelagic piscivorous fishes in the Columbia River plume during spring/early summer 1998–2003: relationships to oceanographic conditions, forage fishes, and juvenile salmonids. Progress in Oceanography. 2006; 68: 1–26.

[20] Peterson WT, Morgan CA, Fisher JP, Casillas E. Ocean distribution and habitat associations of yearling coho (*Oncorhynchus kisutch*) and Chinook (*O*. *tshawytscha*) salmon in the northern California Current. Fisheries Oceanography. 2010; 19: 508–525.

[21] Renner M, Arimitsu ML, Piatt JF. Structure of marine predator and prey communities along environmental gradients in a glaciated fjord. Canadian Journal Fisheries and Aquatic Sciences. 2012; 69: 2029–2045.

[22] Gremillet D, Lewis S, Drapeau L, van Der Lingen CD, Huggett JA, Coetzee JC, et al Spatial match-mismatch in the Benguela upwelling zone: should we expect chlorophyll and sea-surface temperature to predict marine predator distributions? Journal of Applied Ecology. 2008; 45: 610–621.

[23] Thayer JA, Bertram DF, Hatch SA, Hipfner MJ, Slater L, Sydeman WJ, Watanuki Y. Forage fish of the Pacific Rim as revealed by diet of a piscivorous seabird: synchrony and relationships with sea surface temperature. Canadian Journal of Fish and Aquatic Sciences. 2008; 65: 1610–1622.

[24] NOAA (National Oceanic and Atmospheric Administration). West Coast Regional Node home page. 2007. < http://coastwatch.pfel.noaa.gov/index.html >. Accessed July 29, 2015.

[25] Day RH, Alexander KP, Nigro DA. Ecological specialization and overlap of Brachyramphus murrelets in Prince William Sound, Alaska. Auk. 2003; 120: 680–699.

[26] Raphael MG, Shirk AJ, Falxa GA, Pearson SF. Habitat associations of marbled murrelets during the nesting season in nearshore waters along the Washington to California coast. Journal of Marine Systems. 2015; 146: 17–25.

[27] Speckman SG, Piatt JF, Springer AM. Small boats disturb fish-holding marbled murrelets. Northwestern Naturalist. 2004; 85: 32–34.

[28] Bellefleur D, Lee P, Ronconi RA. The impact of recreational boat traffic on marbled murrelets (*Brachyramphus marmoratus*). Journal of Environmental Management. 2009; 90: 531–538. doi: 10.1016/j.jenvman.2007.12.002 18222029 10.1016/j.jenvman.2007.12.002

[29] Halpern BS, Frazier M, Potapenko J, Casey KS, Koenig K, Longo C, et al Spatial and temporal changes in cumulative human impacts on the world’s ocean. Nature Communications. 2015; 6: 7615 doi: 10.1038/ncomms8615 26172980 10.1038/ncomms8615 PMC4510691

[30] Sanderson EW, Jaiteh M, Levy MA, Redford KH, Wannebo AV, Woolmer G. The human footprint and the last of the wild. Bioscience. 2002; 52: 891–904.

[31] Sealy SG. Feeding ecology of the ancient and marbled murrelets near Langara Island, British Columbia. Canadian Journal Zoology. 1974; 53: 418–433.

[32] NREL (National Renewable Energy Laboratory). WINDExchange 50-m wind energy map. 2012. < http://www.nrel.gov/wind/>. Accessed April 10, 2015.

[33] Carter HA. At-sea biology of the marbled murrelet (Brachyramphus marmoratus) in Barkley Sound, British Columbia. MS Thesis. University of Manitoba. 1984.

[34] Yen PPW, Huettmann F, Cooke F. A large-scale model for the at-sea distribution and abundance of marbled murrelets (*Brachyramphus marmoratus*) during the breeding season in coastal British Columbia, Canada. Ecological Modelling. 2004; 171: 395–413.

[35] Barrett J. The influence of oceanographic and terrestrial attributes on marbled murrelet (Brachyramphus marmoratus) marine habitat selection during the breeding season. M. Sc. Thesis, Simon Fraser University. 2008.

[36] NOAA (National Oceanic and Atmospheric Administration). Environmental Sensitivity Index. 2002. < http://response.restoration.noaa.gov/maps-and-spatial-data/environmental-sensitivity-index-esi-maps.html>. Accessed March 3, 2015.

[37] Miller SL, Meyer CB, Ralph CJ. Land and seascape patterns associated with marbled murrelet abundance offshore. Waterbirds. 2002; 25: 100–108.

[38] Kennedy RE, Yang Z, Cohen WB. Detecting trends in forest disturbance and recovery using yearly Landsat time series: 1. LandTrendr–Temporal segmentation algorithms. Remote Sensing of Environment. 2010; 114: 2897–2910.

[39] Ohmann JL, Gregory MJ, Henderson EB, Roberts HM. Mapping gradients of community composition with nearest-neighbor imputation: extending plot data for landscape analysis. Journal of Vegetation Science. 2011; 22: 660–676.

[40] FLNRO (Forests, Lands and Natural Resource Operations). Old growth management areas. British Columbia Ministry of Forests, Lands, and Natural Resource Operations as Old Growth Management Areas. 2012. <https://apps.gov.bc.ca/pub/geometadata/home.do >. Accessed May 22, 2012.

[41] Becker BH, Beissinger SR. Scale-dependent habitat selection by a nearshore seabird, the marbled murrelet, in a highly dynamic upwelling system. Marine Ecology Progress Series. 2003; 256: 243–255.

[42] Peery MZ, Newman SH, Storlazzi CD, Beissinger SR. Meeting reproductive demands in a dynamic upwelling system: foraging strategies of a pursuit-diving seabird, the marbled murrelet. Condor. 2009; 111: 120–134.

[43] Naslund NL. Why do marbled murrelets attend old-growth forest nesting areas year-round? Auk. 1993; 110: 594–602.

[44] Peery MZ, Beissinger SR, Newman SH, Becker BH, Burkett EB, Williams TD. Individual and temporal variation in inland flight behavior of marbled murrelets: implications for population monitoring. Condor. 2004; 160: 344–353.

[45] Barbaree BA, Nelson SK, Dugger BD. Marine space use by marbled murrelets Brachyramphus marmoratus at a mainland fjord system in southeast Alaska. Marine Ornithology. 2015; 116: 173–184.

[46] McFarlane Tranquilla LA, Yen PP-W, Bradley RW, Vanderkist BA, Lank DB, Parker NR, et al Do two murrelets make a pair? Breeding status and behavior of marbled murrelet pairs captured at sea. Wilson Bulletin. 2003; 115: 374–381.

[47] Kuletz KJ, Piatt JF. Juvenile marbled murrelet nurseries and the productivity index. Wilson Bulletin. 1999; 111: 257–261.

[48] Ronconi RA. Patterns and processes of marine habitat selection: foraging ecology, competition, and coexistence among coastal seabirds. PhD dissertation. University of Victoria. 2008.

[49] Beyer HL. Geospatial Modeling Environment. 0.7.1.0. 2012.

[50] Kuletz KJ. Foraging behavior and productivity of a non-colonial seabird, the marbled murrelet (Brachyramphus marmoratus), relative to prey and habitat. P.D. dissertation, University of Victoria. 2005.

[51] Burger AE, Hitchcock CL, Stewart EA, Davoren GK. Coexistence and spatial distributions of marbled murrelets (*Brachyramphus marmoratus*) and other alcids off southwest Vancouver Island, British Columbia. Auk. 2008; 125: 192–204.

[52] Baasch CM, Tyre AJ, Millspaugh JJ, Hygnstrom SE, Vercauteren KC. An evaluation of three statistical methods used to model resource selection. Ecological Modelling. 2010; 221:565–574.

[53] Burnham KP, Anderson DR. Model selection and multimodel inference: a practical information-theoretic approach Springer, New York 2002.

[54] Franklin AB, Anderson DR, Gutierrez RJ, Burnham KP. Climate, habitat quality, and fitness in northern spotted owl populations in northwestern California. Ecological Monographs. 2000; 70: 539–590.

[55] Ralph CJ, Hunt GL, Raphael MG, Piatt JF. Ecology and conservation of the marbled murrelet in North America: an overview. In: Ralph CJ, Hunt GL, Raphael MG, Piatt JF, editors. Ecology and conservation of the marbled murrelet. General Technical Report. PSW-GTR-152. U.S. Department of Agriculture, Forest Service. Pp. 3–22. 1995.

[56] Dormann CF, Elith J, Bacher S, Buchmann C, Carl G, Carre G, et al Collinearity: a review of methods to deal with it and a simulation study evaluating their performance. Ecography. 2013; 32: 27–46.

[57] Johnson JB, Omland KS. Model selection in ecology and evolution. Trends in Ecology and Evolution. 2004; 19: 101–108. 16701236 10.1016/j.tree.2003.10.013

[58] Hosmer DW, Lemeshow S, Sturdivant RX. Applied Logistic Regression Third edition John Wiley and Sons, Inc Hoboken, NJ 2013.

[59] Bonnot TW, Millspaugh JJ, Rumble MA. Multi-scale nest-site selection by black-backed woodpeckers in outbreaks of mountain pine beetles. Forest Ecology and Management. 2009; 259: 220–228.

[60] Boyce MS, Vernier PR, Nielsen SE, Schmiegelow FKA. Evaluating resource selection functions. Ecological Modelleing. 2002; 157: 281–300.

[61] R Development Core Team. R: A language and environment for statistical computing. Vienna, Austria: R Foundation for Statistical Computing. 2014.

[62] Hebert PN, Golightly RT. At-sea distribution and movements of nesting and non-nesting marbled murrelets *Brachyramphus marmoratus* in northern California. Marine Ornithology. 2008; 36: 99–201.

[63] Raphael MG. Conservation of the marbled murrelet under the Northwest Forest Plan. Conservation Biology. 2006; 20: 297–305. 16903091 10.1111/j.1523-1739.2006.00382.x

[64] Raphael MG, Mack DE, Cooper BA. Landscape-scale relationships between abundance of marbled murrelets and distribution of nesting habitat. Condor. 2002; 104: 331–342.

[65] Bailey H, Brookes KL, Thompson PM. 2014 Assessing environmental impacts of offshore wind farms: lessons learned and recommendations for the future Aquatic Biosystems 10:1–13. 25250175 10.1186/2046-9063-10-8 PMC4172316

[66] Pinto JM, Pearson WH, Anderson JW. Sediment preferences and oil contamination in the Pacific sand lance (*Ammodytes hexapterus*). Marine Biology. 1984; 83: 193–204.

[67] Robards MD, Piatt JF, Rose GA. Maturation, fecundity, and intertidal spawning of Pacific sand lance in the northern Gulf of Alaska. Journal of Fish Biology. 1999; 54: 1050–1068.

[68] Haynes TB, Ronconi RA, Burger AE. Habitat use and behavior of the Pacific sand lance (*Ammodytes hexapterus*) in the shallow subtidal region of southwestern Vancouver Island. Northwestern Naturalist. 2007; 88: 155–167.

[69] Haynes TB, Robinson C.K.L., Dearden P. Modelling nearshore intertidal habitat use of young-of-the-year Pacific sand lance (*Ammodytes hexapterus*) in Barkley Sound, British Columbia, Canada. Environmental Biology of Fishes. 2008; 83: 487–498.

[70] Bertram DF, Kaiser GW, Ydenberg RC. Patterns in the provisioning and growth of nestling rhinocerous auklets. Auk. 1991; 108: 842–852.

[71] Gjerdrum C, Vallee AMJ, St Clair CC, Bertram DF, Ryder JL, Blackburn GS. Tufted puffin reproduction reveals ocean climate variability. Proceedings of the National Academy of Sciences. 2003; 100: 9377–9382. 10.1073/pnas.1133383100 PMC170926 12871995

[72] Hedd A, Bertram DF, Ryder JL, Jones IL. Effects of inter-decadal climate variability on marine trophic interactions: rhinoceros auklets and their fish prey. Marine Ecology Progress Series. 2006; 309: 263–278.

[73] Selleck JR, Gibson CF, Shull S, Gaydos JK. Nearshore distribution of Pacific sand lance (*Ammodytes personatus*) in the inland waters of Washington State. Northwestern Naturalist. 2016; 96: 185–195.

[74] Hazlitt SL, Martin TG, Sampson L, Arcese P. The effects of including marine ecological values in terrestrial reserve planning for a forest-nesting seabird. Biological Conservation. 2010; 143: 1299–1303.

[75] Withey JC, Bloxton TD, Marzluff JM. Effects of tagging and location error in wildlife radiotelemetry studies In: Millspaugh JJ, Marzluff JM, editors. Radio tracking and animal populations. San Diego, CA: Academic Press; 2001 Pp. 43–75.

[76] Rettie WJ, McLoughlin PD. Overcoming radiotelemetry bias in habitat-selection studies Canadian Journal of Zoology. 1999; 77: 1175–1184.

[77] Meyer CB, Miller SL, Ralph CJ. Multi-scale landscape and seascape patterns associated with marbled murrelet nesting areas on the U.S. west coast. Landscape Ecology. 2002; 17: 95–115.

[78] Boyce MS. Scale for resource selection functions. Diversity and Distributions. 2006; 12: 269–276.

[79] Fortin D, Beyer HL, Boyce MS, Smith DW, Duchesne T, Mao JS. Wolves influence elk movements in Yellowstone National Park. Ecology. 2005; 86: 1320–1330.

[80] Roever CL, Beyer HL, Chase MJ, van Aarde RJ. The pitfalls of ignoring behavior when quantifying habitat selection. Diversity and Distributions. 2014; 20: 332–333.

[81] Ronconi RA, Burger AE. Limited foraging flexibility: increased foraging effort by a marine predator does not buffer against scarce prey. Marine Ecology Progress Series. 2008; 366: 245–258.

[82] Becker BH, Peery MZ, Beissinger SR. Ocean climate and prey availability affect the trophic level and reproductive success of the marbled murrelet, an endangered seabird. Marine Ecology Progress Series. 2007; 329: 267–279.

